# Functional and Aesthetic Rehabilitation of an Endodontically Treated Tooth Using a Custom Cast Post and Core: A Case Report

**DOI:** 10.7759/cureus.101854

**Published:** 2026-01-19

**Authors:** Pratiksha A Srivastava, Aditya Sharma, Reetika Singh, Jyoti Verma, Sahil Sethi, Apermita Saxena

**Affiliations:** 1 Prosthodontics, Seema Dental College and Hospital, Rishikesh, IND; 2 Periodontics, Seema Dental College and Hospital, Rishikesh, IND; 3 Dentistry, Autonomous State Medical College, Amethi, IND; 4 Prosthodontics, Swami Vivekanand Subharti Dental College and Hospital, Meerut, IND; 5 Prosthodontics, Kalka Dental College, Meerut, IND; 6 Oral Medicine and Radiology, Private Practice, Nakodar, IND

**Keywords:** crown rehabilitation, custom cast post, endodontically treated tooth, post and core, prosthodontic rehabilitation

## Abstract

Endodontically treated teeth often exhibit significant loss of tooth structure, resulting in reduced mechanical strength and increased susceptibility to fracture. Post and core restorations play a crucial role in providing retention for the definitive coronal restoration and in reinforcing the remaining tooth structure. Among the various post systems available, custom cast post and core restorations offer superior adaptation to the root canal morphology, particularly in teeth with extensive coronal destruction or irregular canal configurations. Their ability to provide adequate retention, resistance to functional forces, and long-term durability has been well documented. Appropriate selection of post systems based on remaining tooth structure, canal anatomy, and functional demands is essential for achieving predictable clinical outcomes. This case report illustrates the successful functional and aesthetic rehabilitation of an endodontically treated tooth using a custom cast post and core. It highlights the continued clinical relevance of custom cast post and core systems in selected cases despite the increasing popularity of prefabricated fiber posts.

## Introduction

Endodontic procedures are associated with structural changes in teeth, including reduced elasticity and increased susceptibility to fracture [1,2]. As a consequence of this structural weakening, it becomes essential to provide both strong internal and external support to maintain the integrity and functionality of the tooth. This support is typically achieved through the use of a post and core system [3]. Contemporary dentistry offers multiple post options, including prefabricated fiber posts, customized fiber posts, computer-aided design (CAD)/computer-aided manufacturing (CAM)-fabricated posts, and conventional custom cast post and core restorations, each with specific indications and limitations. 

Custom cast post and core restorations, although well established, continue to be indicated in selected cases requiring precise adaptation to complex canal morphology and fabrication of a single-unit post-core complex [4]. This precise adaptation ensures a snug fit against the canal walls, which enhances the stability of the tooth [5]. Moreover, custom posts are designed to minimize the presence of voids, thereby eliminating potential areas that can compromise the integrity of the restoration [6]. In addition, the use of custom posts allows for a more uniform application of luting cement across the restoration, which significantly contributes to the strength and durability of the outcome [7]. This meticulous attention to detail in the design and placement of custom posts ultimately leads to a higher-quality restoration that better withstands functional forces and promotes long-term tooth preservation. Although fiber-reinforced posts are widely used due to their aesthetic advantages, custom cast post and core restorations continue to be indicated in cases with extensive coronal destruction and unfavorable canal anatomy.

In this context, the present case report illustrates the clinical rationale and short-term outcome of restoring a structurally compromised maxillary canine using a custom cast post and core, emphasizing biomechanical considerations and individualized treatment planning in a functionally demanding clinical scenario.

## Case presentation

**Figure 1:**
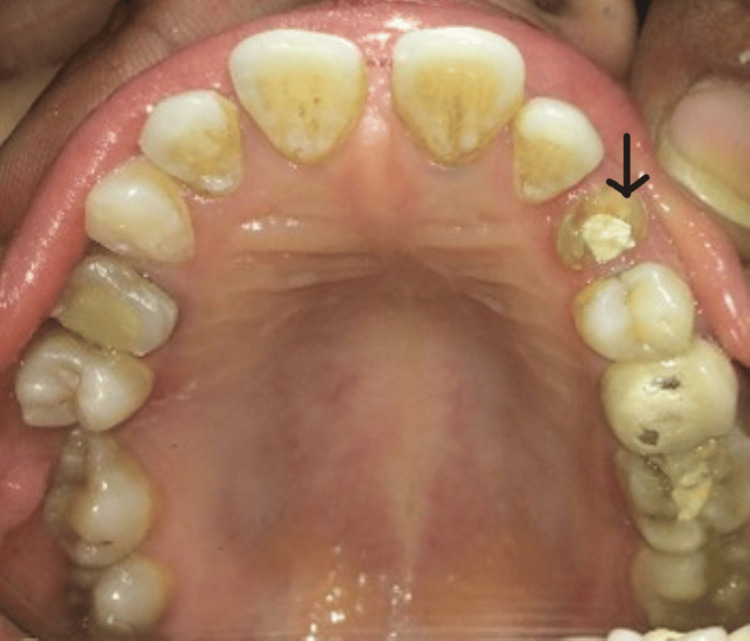
Clinical preoperative view revealed a temporary restoration at #23 (arrow).

A 35-year-old female patient was referred from the department of endodontics after undergoing endodontic restoration of her maxillary left canine for a post and core procedure. The patient reported a history of intermittent pain and swelling that began two weeks after her previously placed crown was dislodged. The patient reported a history of root canal treatment followed by crown placement one year earlier due to dental caries. Following crown dislodgement, secondary infection developed, necessitating retreatment. Therefore, a re-root canal treatment was performed. Subsequently, the patient's medical history was taken, which was not relevant. On clinical examination, a prepared maxillary left canine with a temporary restoration was observed (Figure 1). Periodontal examination revealed healthy surrounding tissues with no signs of active periodontal disease. Radiographic examination confirmed the status of the endodontically treated tooth. It demonstrated satisfactory obturation following retreatment, adequate root length, and absence of periapical pathology. Considering the tooth involved was a maxillary canine subjected to significant lateral functional loads, along with extensive coronal tooth structure loss and an irregular canal morphology, a custom cast post and core was selected to provide enhanced resistance to rotational forces and predictable core stability. Written informed consent was obtained from the patient, and treatment was initiated.

Ethical approval for this case report was obtained from the institutional ethics committee of Seema Dental College and Hospital, and the study was conducted in accordance with the ethical standards laid down in the Declaration of Helsinki.

Procedure

Clinically, the remaining coronal tooth structure permitted the establishment of a ferrule through conservative tooth preparation without the need for additional periodontal procedures, and the tooth was subjected to lateral functional forces due to its role in canine guidance. These factors collectively influenced the decision to restore the tooth using a custom cast post and core.

The preparation for the post began by placing a hot endodontic plugger (Dentsply Sirona, Germany) approximately half the length of the canal to remove gutta-percha. This was followed by the actual dowel preparation using peeso reamers (Dentsply Maillefer, Ballaigues, Switzerland). Its sharp, non-cutting tip followed the path of least resistance, which was the gutta-percha in the canal. The peeso reamer was measured against the radiograph of the tooth to determine the length to which the reamer would be inserted into the canal.

**Figure 2:**
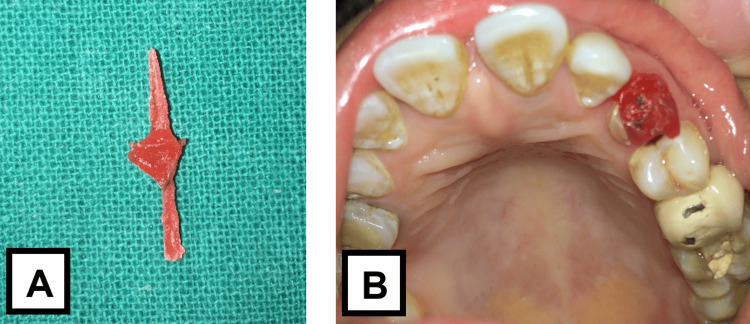
(A) Impression (with handle) made with the help of pattern resin using direct technique. (B) Fit of the impression (without handle) evaluated intraorally. A small rod of Pattern resin was pre-made to take an impression over it using the direct technique.

**Figure 3:**
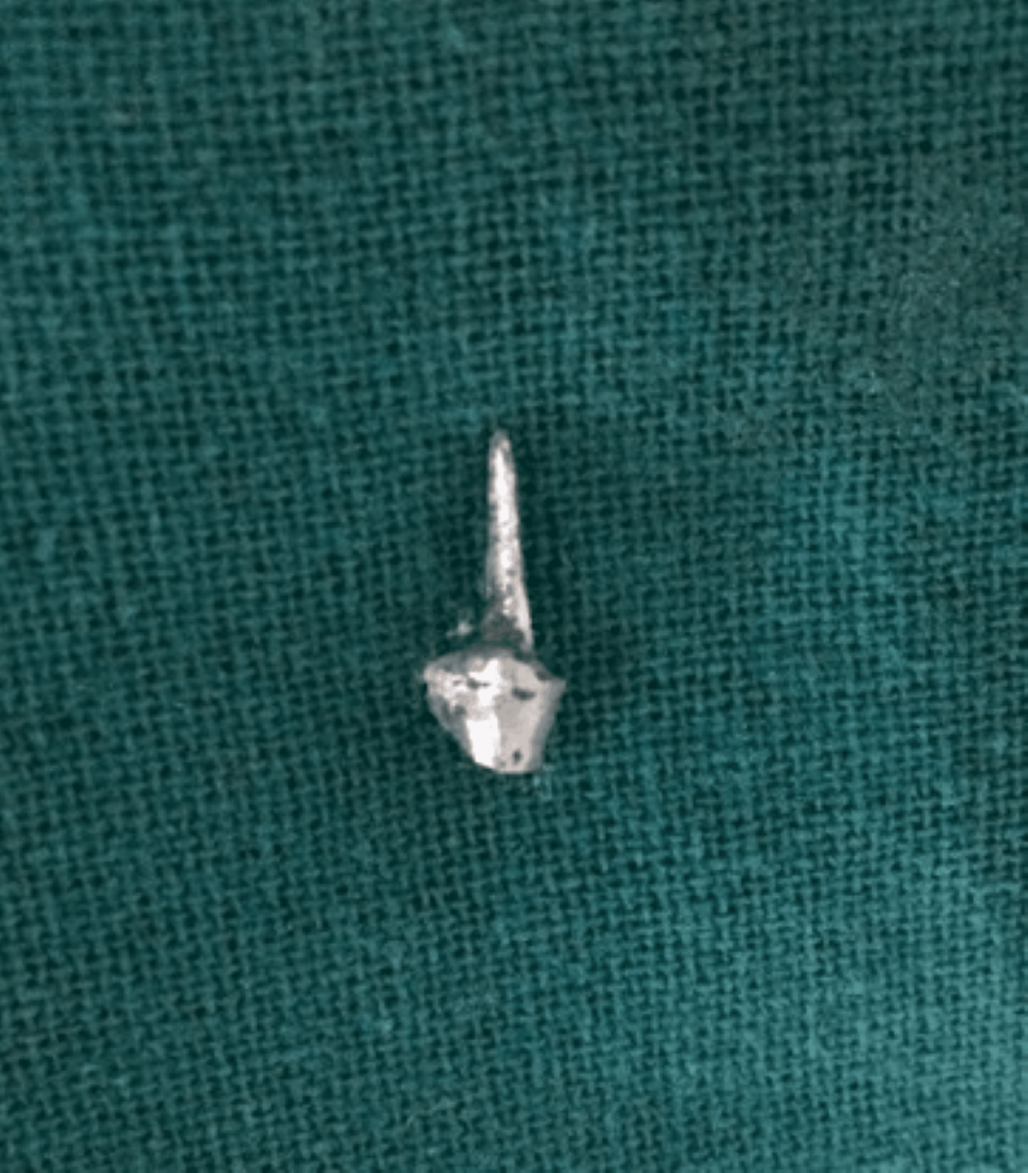
Custom cast post and core. The pattern resin was cast in cobalt-chromium alloy to produce cast post and core.

**Figure 4:**
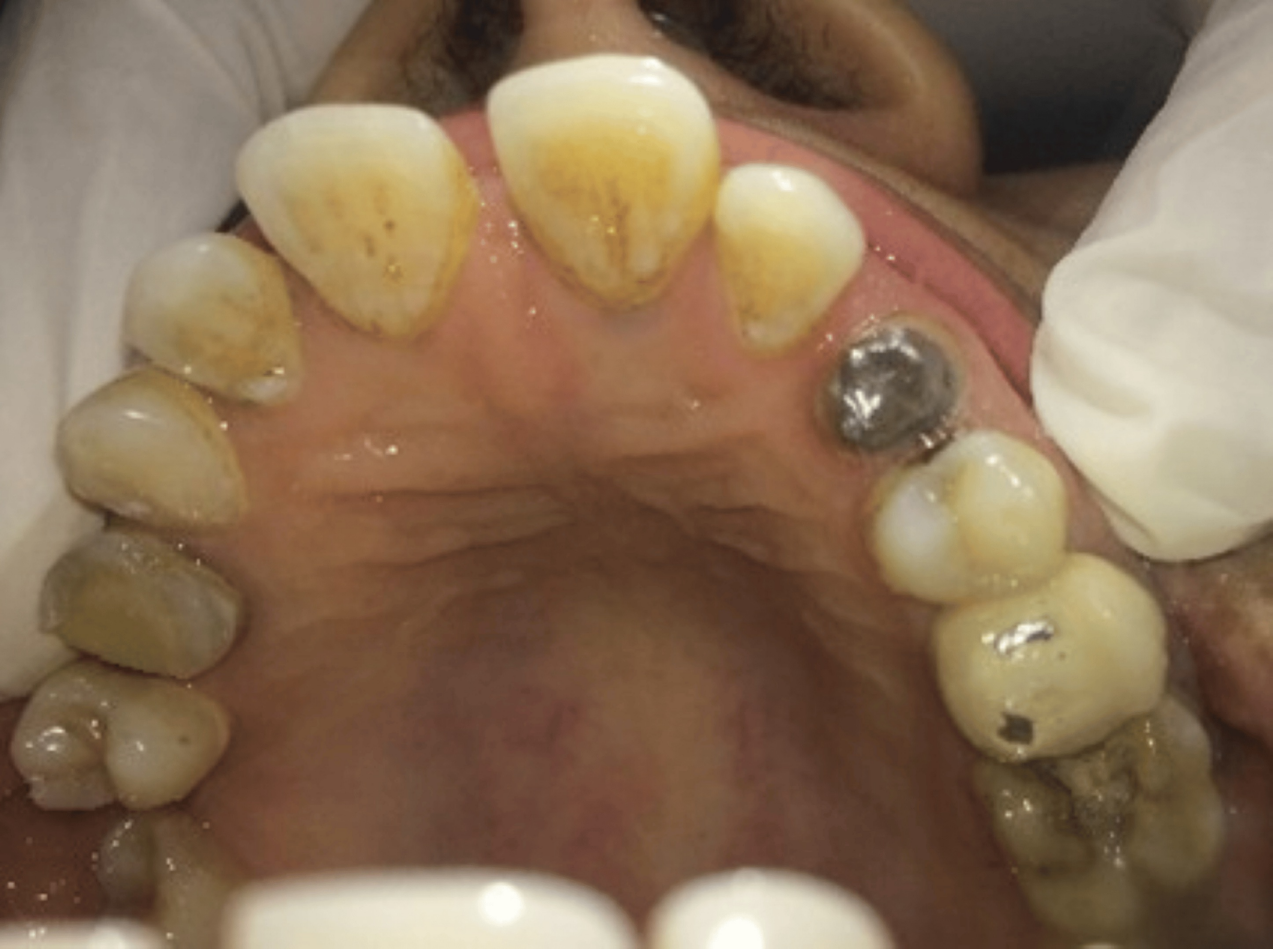
Clinical view of cast post and core cemented. The customised cast post and core were cemented using type I GIC. GIC: glass ionomer cement

**Figure 5:**
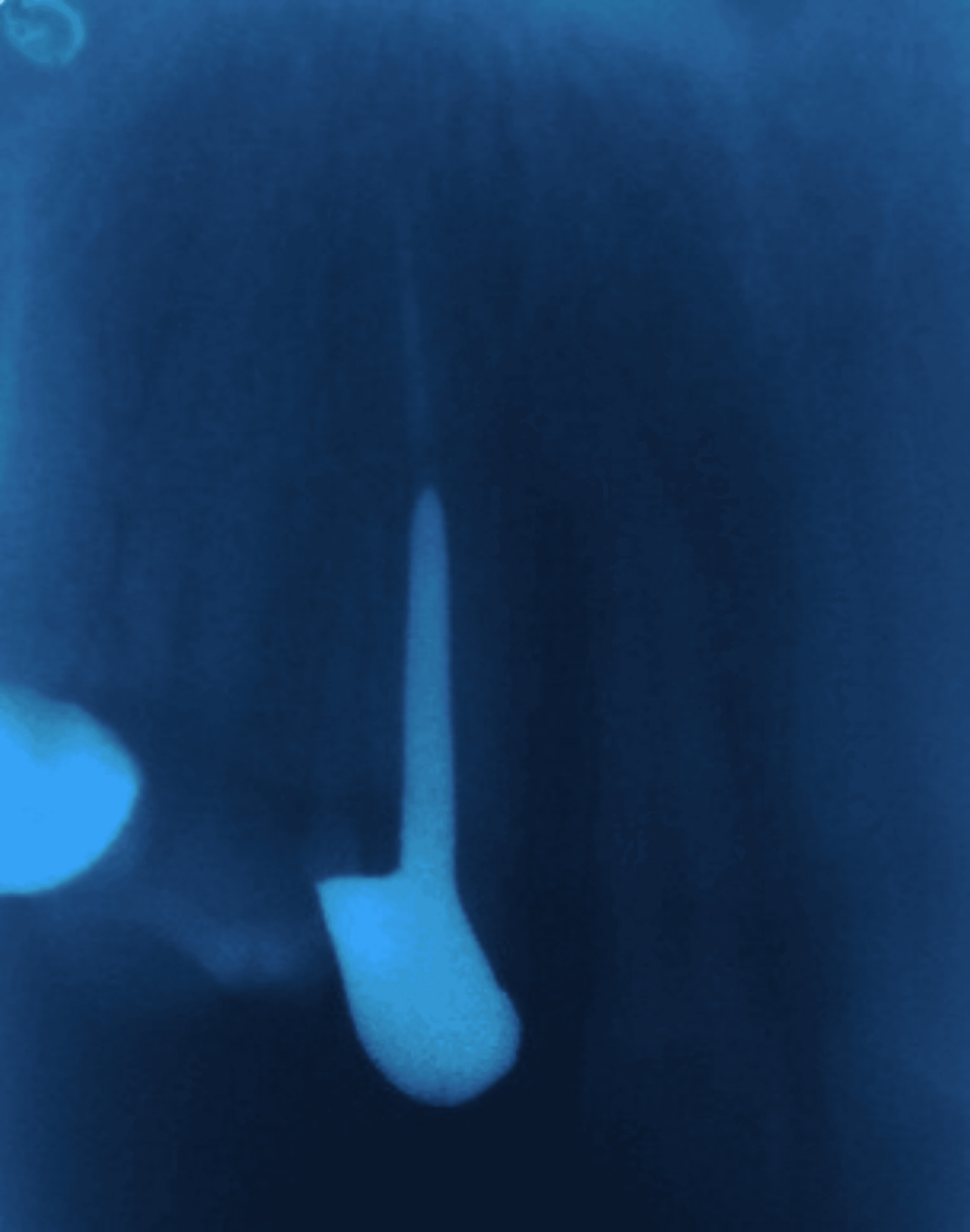
Radiographic view of cast post and core cemented.

Post space preparation was completed using sequential peeso reamers up to a diameter of 1.5 mm, maintaining adequate apical gutta-percha. A keyway was placed in the orifice of the canal to provide anti-rotational stability to the dowel. A prominent contrabevel was added to remove unsupported enamel to provide a collar around the occlusal circumference of the preparation. This aids in holding the tooth together and helps prevent fracture. It also serves as a safeguard when using a precision-fitting dowel, which can exert lateral forces during cementation. A wide, distinct bevel was placed around the occlusal external periphery of the preparation with a flame diamond (MANI, Inc., Japan). Subsequently, a direct post and core pattern was fabricated using pattern resin (EZ-Pattern, Hudens Bio, South Korea) (Figure 2), selected for its dimensional stability and ease of handling during direct pattern fabrication. The pattern was then invested and cast into a customized cast post and core (Figure 3), designed to provide optimal retention and support for the final restoration. After thorough try-in and adjustments, the cast post and core were cemented using type I glass ionomer cement (GIC) (GC Corporation, Tokyo, Japan) (Figures 4-5), selected for its chemical adhesion to dentin, favorable handling properties, and long-term clinical performance in cast post cementation.

**Figure 6:**
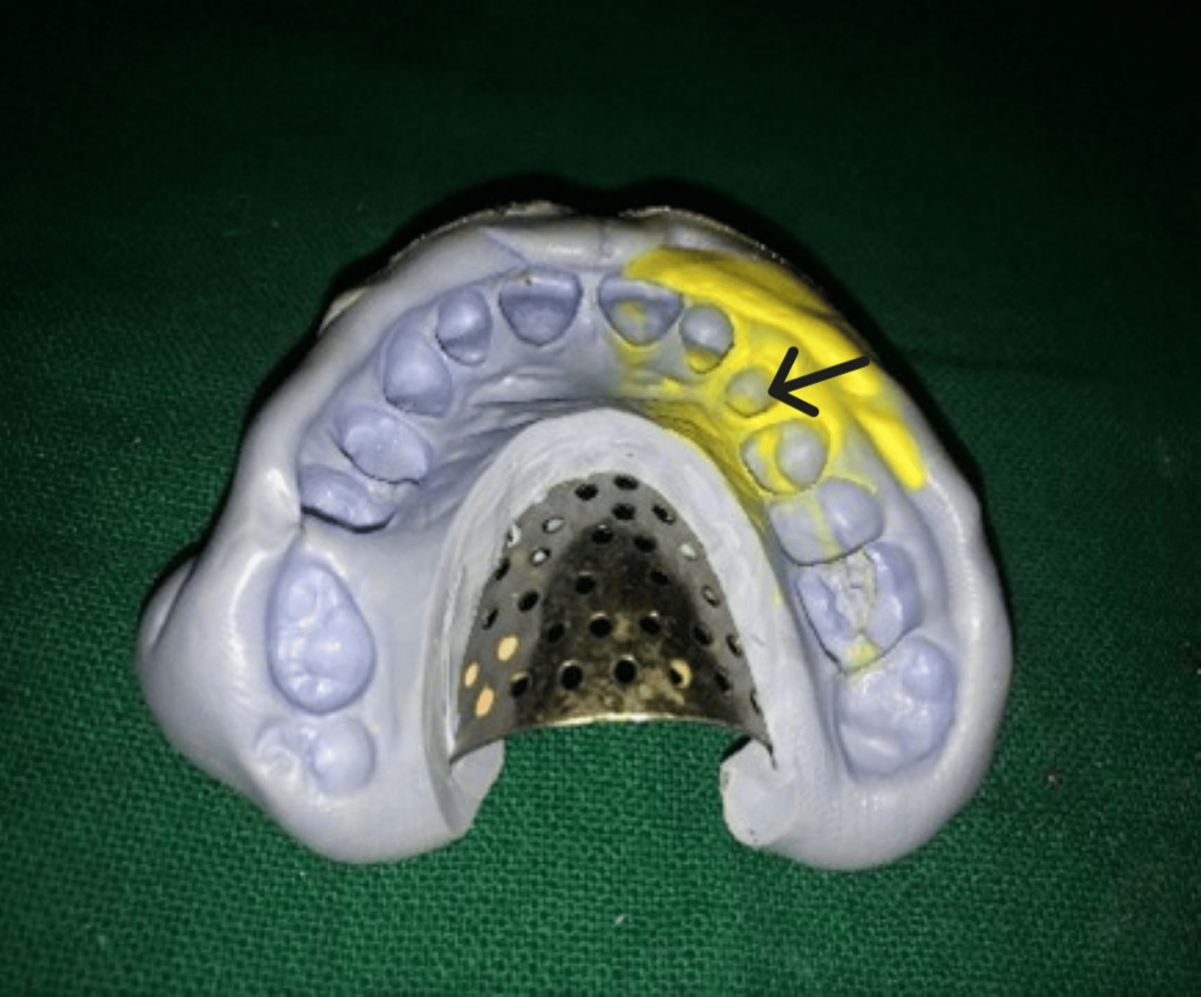
Elastomeric impression for crown with respect to #23 (arrow). A putty and light body impression was made with respect to #23 using a stock tray.

**Figure 7:**
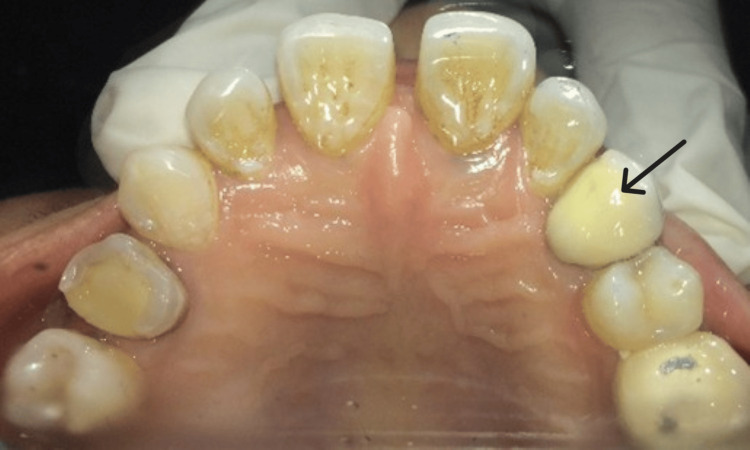
PFM crown (arrow) cemented. PFM crown was luted with type I GIC. PFM: porcelain-fused-to-metal; GIC: glass ionomer cement

Following successful cementation, a detailed impression of the prepared tooth was made to facilitate the precise fabrication of the porcelain-fused-to-metal (PFM) crown (Figure 6). Once the crown was fabricated and verified for fit and aesthetics, it was cemented into position (Figure 7), restoring both function and appearance to the tooth.

**Figure 8:**
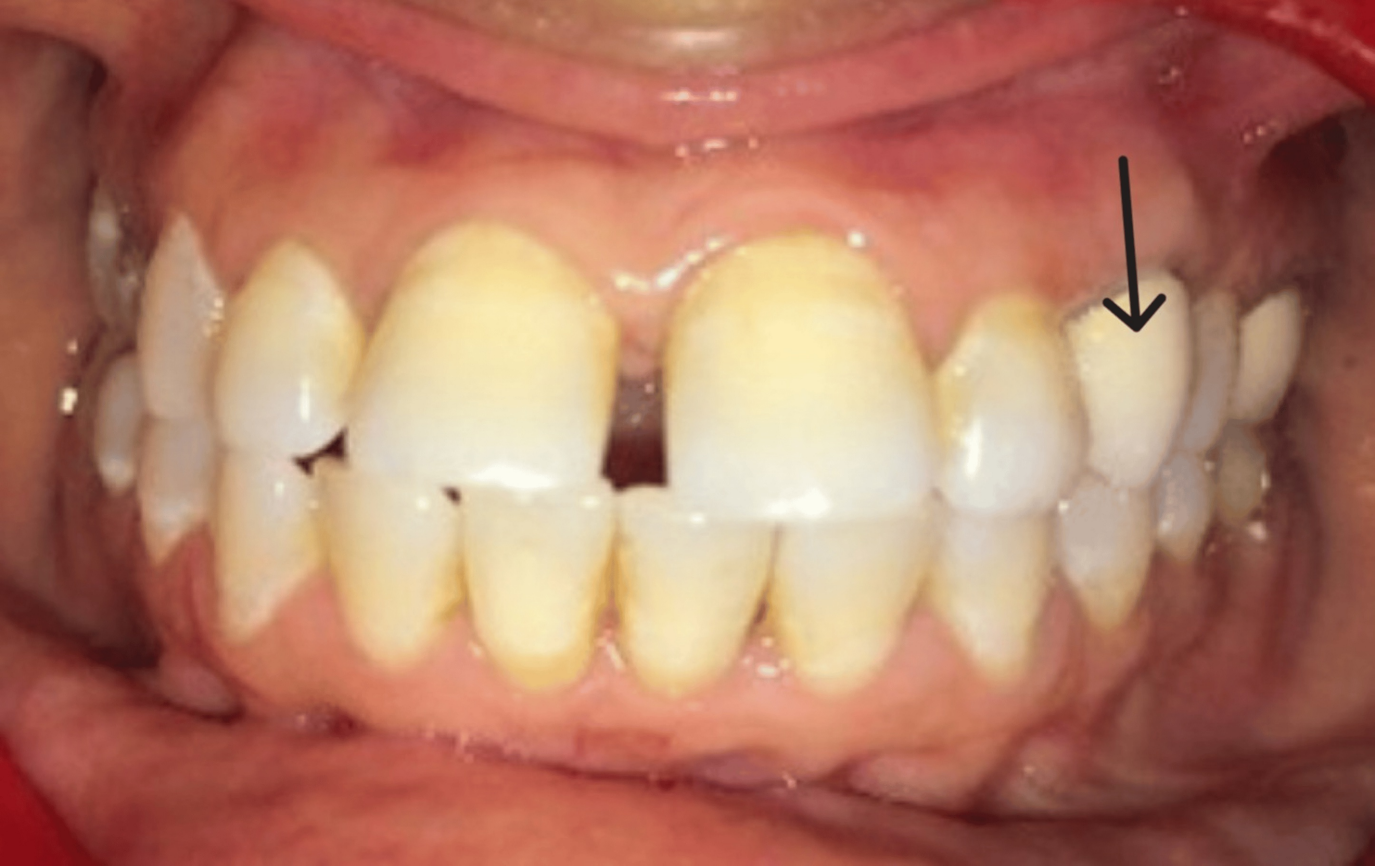
Clinical view of crown (arrow) at one month follow-up visit.

The patient was reviewed after one week and one month (Figure 8) following crown cementation. At follow-up visits, the patient reported no pain or discomfort. Clinical examination revealed satisfactory crown retention, absence of mobility, and healthy surrounding periodontal tissues with no signs of inflammation. The restoration was functionally stable, and the patient expressed satisfaction with both aesthetics and function. The short-term prognosis of the restored tooth was considered favorable. 

## Discussion

Restoration of endodontically treated teeth with extensive coronal destruction remains a clinical challenge when the remaining tooth structure is insufficient to support a definitive restoration. In such situations, the post and core procedure is essential to provide retention and resistance for the final prosthesis and to restore structural integrity and function [8-10].

Among the various post systems available, custom cast post and core restorations continue to be indicated in teeth with minimal residual coronal structure and complex canal morphology. Their ability to precisely adapt to the internal configuration of the root canal allows fabrication of a single-unit post-core complex with predictable retention, particularly in oval or elliptical canals where prefabricated posts may be inadequate [11-13]. Additionally, the possibility of modifying core angulation makes cast posts advantageous when alignment correction is required for optimal prosthetic rehabilitation [14].

Long-term clinical studies have demonstrated favorable survival outcomes for cast post and core restorations when appropriate case selection and biomechanical principles are followed. Gómez-Polo et al. reported high survival rates exceeding 10 years, while Creugers et al. and Ellner et al. confirmed predictable clinical performance over extended follow-up periods [15-18]. These findings support the continued relevance of cast posts in contemporary restorative practice.

Maxillary canines present unique biomechanical considerations due to their role in canine guidance and exposure to significant lateral and oblique functional forces. In the present case, severe coronal destruction combined with functional demands limited the predictability of adhesive-dependent post systems. A custom cast post and core was therefore selected to achieve optimal canal adaptation, resistance to rotational forces, and controlled core angulation.

Despite these advantages, cast post and core restorations are associated with important limitations. Owing to their high elastic modulus relative to dentin, cast posts may concentrate stresses within the root, producing a wedging effect under functional loading. This can predispose the tooth to unfavorable vertical or oblique root fractures, which are often non-restorable and associated with a poor long-term prognosis. Additional drawbacks include increased chairside and laboratory time, cost, and aesthetic concerns in anterior teeth. These limitations, along with the advent of fiber-reinforced post systems and advanced CAD/CAM systems with favorable stress distribution and aesthetic properties, have contributed to a decline in the use of cast posts [19]. However, the present case report illustrates the choice of a custom cast post and core guided by the need to manage a structurally compromised maxillary canine subjected to functional loading, where anatomical constraints, biomechanical considerations, and restorative requirements favored this approach despite the availability of newer post systems.

## Conclusions

A wide range of post systems is available for restoring endodontically treated teeth, making it essential for clinicians to understand their indications, advantages, and limitations. Although fiber posts are popular and there have been recent advancements, traditional custom cast systems still remain clinically relevant in selected prosthodontic rehabilitations. The indication for a cast post and core should be guided by careful case selection, taking into account biomechanical demands, canal anatomy, and the extent of remaining coronal tooth structure. The present case highlights the importance of individualized treatment planning in achieving predictable clinical outcomes. As a single case report, the findings cannot be generalized, and long-term comparative studies are required to further define the role of cast post and core systems in modern restorative dentistry.
